# Metabolic regulation of T cell development

**DOI:** 10.3389/fimmu.2022.946119

**Published:** 2022-07-25

**Authors:** Mengdi Zhang, Xiaoxi Lin, Zhou Yang, Xia Li, Zhiguang Zhou, Paul E. Love, Jiaqi Huang, Bin Zhao

**Affiliations:** ^1^ National Clinical Research Center for Metabolic Diseases, Key Laboratory of Diabetes Immunology, Ministry of Education, and Department of Metabolism and Endocrinology, The Second Xiangya Hospital of Central South University, Changsha, China; ^2^ Section on Hematopoiesis and Lymphocyte Biology, Eunice Kennedy Shriver National Institute of Child Health and Human Development, National Institutes of Health, Bethesda, MD, United States

**Keywords:** T cell development, thymocytes, T cell metabolism, thymus, thymocyte metabolism

## Abstract

T cell development in the thymus is tightly controlled by complex regulatory mechanisms at multiple checkpoints. Currently, many studies have focused on the transcriptional and posttranslational control of the intrathymic journey of T-cell precursors. However, over the last few years, compelling evidence has highlighted cell metabolism as a critical regulator in this process. Different thymocyte subsets are directed by distinct metabolic pathways and signaling networks to match the specific functional requirements of the stage. Here, we epitomize these metabolic alterations during the development of a T cell and review several recent works that provide insights into equilibrating metabolic quiescence and activation programs. Ultimately, understanding the interplay between cellular metabolism and T cell developmental programs may offer an opportunity to selectively regulate T cell subset functions and to provide potential novel therapeutic approaches to modulate autoimmunity.

## Introduction

T cell development is tightly regulated by multiple checkpoints and proliferative events before the emergence of naive T cells from the thymus (1–3). Early thymic progenitors (ETPs), also known as double-negative 1 (DN1) cells, differentiate into DN2 thymocytes in the thymic parenchyma, gaining T-lineage commitment (4, 5). A paramount event in T cell development called β-selection occurs during the DN3 stage, and thymocytes that successfully assemble the pre-T cell receptor (TCR) hasten to the DN4 phase and initiate rapid cell cycling governed by complex regulatory metabolism (6, 7). Additionally, γδ and αβ T cell lineages diverge at the DN3 stage (8, 9). Thymocytes return to a quiescence state in double-positive (DP) stage, undergoing positive/negative selection and DP to single-positive (SP) transition before becoming mature CD4+ or CD8+ T cells (3, 10, 11).

Cellular metabolism integrates multiple pathways and large networks of chemical reactions, and plays a critical role in regulating almost all cellular processes (12). Different thymocyte subsets have distinct metabolic patterns tailored to meet the bioenergetic demands required at each stage (13–15). In summary, catabolic metabolism of amino acids and glucose promotes the energy and biosynthesis that quiescent thymocytes require, whereas the transition from resting cells into highly activated phenotypes requires substantial metabolic reprogramming comprising aerobic glycolysis (Warburg effect), glutaminolysis, and mitochondrial biogenesis, which expedites oxidative phosphorylation (OXPHOS) and one-carbon metabolism (10, 15–17). These metabolic alterations constitute complex signaling mechanisms that connect external signals with transcriptional events and fate verdicts (17, 18). Here, we summarize recent findings on the metabolic control of T cell development, and highlight the roles of cell-intrinsic and cell-extrinsic metabolic factors involved in these processes.

## Thymocytes are relatively quiescent before β-selection

ETPs settling in the thymus are quiescent before the first run of proliferation (11). Cytokines, such as Interleukin-7 (IL-7), Kit, and C-X-C motif chemokine receptor 4 (CXCR4), as well as Notch and Wnt signaling, account for the proliferation of thymocytes before β-selection (6, 19–29). During the four DN stages, Notch and IL-7 signaling drive the maturation of thymocytes and regulate cellular metabolism by interacting with the phosphatidylinositol-3-kinase/protein kinase B/mammalian target of rapamycin (PI3K/Akt/mTOR) signaling pathway (18, 21, 30–38). Before accelerating the multiplication at the DN3b stage, thymocytes are quiescent and primarily rely on OXPHOS to maintain bioenergetic homeostasis. AMP-activated protein kinase (AMPK) is a major regulator of metabolism that senses bioenergetic undulation and maintains energy homeostasis in cells (39–41). AMPK works in concert with liver kinase B1 (LKB1) to suppress biosynthesis and energy production, such as glycolysis and lipid synthesis, to restrain the anabolic growth of thymocytes (41–43). Loss of LKB1 leads to thymocyte developmental block and reduction of peripheral T cells (44, 45). Fatty acid-binding protein 3 (FABP3) regulates cellular lipid metabolism by binding to polyunsaturated fatty acids (PUFAs), such as ω-6 PUFAs. Recently, it has been shown that loss of FABP3 redirects DN2 thymocytes to pathogenic Vγ4+ γδ T cells (46). Mitochondrial metabolism also plays a role in thymocyte development. For example, the Janus mitochondrial protein apoptosis-inducing factor (AIF) has been shown to affect thymocyte transitions from DN1 to DN4 by modulating mitochondrial metabolism (47).

## β-selected thymocytes exhibit robust cell proliferation and metabolic reprogramming

Transition from the DN3 to the immature single-positive (ISP) phase, thymocytes undergo V(D)J rearrangement and β-selection. This exclusive selection event then gives rise to robust cell growth and proliferation in which cells have to thoroughly alter their metabolism to meet the increased energy demand. Energy generation by cycling thymocytes after β-selection is largely dependent on aerobic glycolytic metabolism for prioritizing efficient and rapid biosynthesis of intracellular constituents, including nucleic acids and lipids (3, 11). The metabolic switch is mainly triggered by pre-TCR signaling (6, 48–50). Signaling of pre-TCR, Notch, and IL-7 converges to activate PI3K signaling, which stimulates the transition to anabolic metabolism, especially glycolysis. Glucose transporter type 1 (Glut1**)**, an important glucose transporter, is induced at these phases, and its expression level is dependent on the activation of PI3K-Akt signaling (51–54).

The PI3K-phosphoinositol-dependent protein kinase-1 (PDK1)-Akt axis plays a critical role in thymocyte maturation (55, 56). In addition to their well-described role in protein synthesis *via* mTOR signaling, PI3K dominates aerobic glycolysis and glucose metabolism in a variety of biological processes (57, 58). PDK1 regulates the expression of key amino acid and iron transporters and controls the switch of glucose metabolism from aerobic oxidation to glycolysis in the thymus (55, 59). Loss of PDK1 impairs nutrient receptor expression and hence renders metabolically deficient to meet the energy demands from the DN to DP stage transition (55, 60). Furthermore, Akt signaling is a major stimulus of anabolism (21, 61, 62). Deletion of Akt alters thymocyte subsets with a development blocked after DN3 stage (56, 63). Similar to AKT, PIM kinases are also linchpins that regulate the expansion of thymocytes undergoing β-selection (64–66). Of note, the lipid kinase inositol-trisphosphate 3-kinase B (Itpkb) affects β-selection by restricting metabolic activation in DN3 thymocytes. The deficiency of Itpkb leads to accelerated development from DN3 cells to DP cells by activating Akt-mTOR signaling and breaking the balance between Notch and pre-TCR signaling (67).

As a master regulator of cell growth and metabolism, mTOR regulates multiple metabolic pathways, such as glutaminolysis, glycolysis, mitochondrial biogenesis, and protein synthesis (68, 69). It has been shown that mTOR signaling regulates thymocyte proliferation, anabolism, and development *via* integrated signals from TCRs, costimulatory molecules, cytokines, and nutrients (70–72). mTOR forms two structurally and functionally distinct complexes, mTOR complex 1 (mTORC1) and mTORC2 (71). Specifically, mTORC1 could integrate TCR and Notch signaling and induce the expression of transcription factors such as cellular myelocytomatosis oncogene (c-Myc) and Sterol-regulatory element binding proteins (SREBPs) for lipid synthesis and ROS production (71, 73). It has been reported that mTORC1 is involved in the reciprocal development of two fundamentally distinct T cell lineages, αβ and γδ T thymocytes (70). Loss of Raptor-mediated mTORC1 activity impairs the development of αβ T cells but promotes γδ T cell generation (70, 74). In addition, hypoxia-inducible factor 1-α **(**HIF1α**)** induced by mTORC1 signaling could increase glycolysis metabolism and the pentose phosphate pathway by controlling the production of glycolytic cascade members such as hexokinase 2 (HK2) and PDK1 (75–78). On the other hand, mTORC2 regulates glycolysis by activating Akt (61, 63, 79). Deletion of the mTORC2 component Rictor leads to thymocyte developmental blocks at the ISP phase, resulting in a reduction of DP cells (80, 81). Thymocyte specific ablation of Sin1, an important component of mTORC2, leads to developmental block at the DN3 to DN4 transition due to impaired proliferation and reduced expression of the glycolytic enzyme pyruvate kinase M2 (PKM2) through mTORC2-peroxisome proliferator-activated receptor γ (PPAR-γ)-PKM2 axes (82, 83). Taken together, the distinct regulation of thymocyte metabolism between mTORC1 and mTORC2 warrants further biological validation.

Myc expression is transcriptionally induced in thymocytes to facilitate the developmental progression from the DN to the DP stage (84–87). As Myc downstream target genes, the thioredoxin-1 (Trx1) system is a biosensor of glucose and energy metabolism that maintains cellular redox balance. Recent data from an animal study have shown that deletion of thioredoxin reductase-1 (Txnrd1), which is critical for the last step of nucleotide biosynthesis, precludes the expansion of DN cells (88). c-Myc gene expression is also regulated by bromodomain protein 4 (BRD4), which is a transcriptional and epigenetic regulator with functions throughout the cell cycle for proliferative regulation (89–93). It has been shown that BRD4 governs the development and differentiation of ISP thymocytes by modulating metabolic pathways and cell cycle progression (94). Deletion of BRD4 in ISP cells leads to a block in the transition to the DP phase, as well as inhibition of glycolysis (94). The mitochondrial protein Optic atrophy 1 (Opa1) was demonstrated to regulate OXPHOS and was required for thymocyte development during β-selection at the DN3 stage (37). The absence of Opa1 damages cellular respiration and induces apoptotic cell death. Mothe-Satney and collaborators found that overexpression of the transcription factor peroxisome proliferator-activated receptor β (PPARβ) increases fatty acid oxidation instead of glucose oxidation, hence restricting the expansion of DN4 cells (95). Furthermore, Zhao et al. demonstrated that PPARβ regulates the expression of the key genes and enzymes in glycolysis, oxidative phosphorylation, and lipogenesis in β-selected thymocytes. PPARβ^mut^ mice exhibited a reduction in thymocyte cell number starting at the DN4 stage (96). Recent laboratory work demonstrated that depletion of mitochondrial pyruvate carrier (MPC) 1, an MPC transporter subunit responsible for moving pyruvate into mitochondria, led to impaired β-selection and decreased αβT cells due to upregulated glycolysis and reduced OXPHOS (97). MicroRNAs and other noncoding RNAs have also been documented to be involved in T cell development as regulators of anabolism and catabolism (98–103). For example, as an important regulator of PI3K, miR-181 has been shown to regulate T cell development, including conventional and unconventional T cells (104–108). The transition from the DN to DP stage was severely impaired in miR-181-deficient mice (109). Single-cell RNA data showed that key genes of the glycolytic, pentose phosphate, and nucleotide biosynthetic pathways were deregulated in miR-181a1b1-deficient DP cells (104). Furthermore, miR-146a, a suppressor of nuclear factor-kappa B (NF-κB) signaling, participates in the regulation of thymocyte positive selection and amino acid metabolism (110).

Metabolic programming has been proposed to govern distinct immune cell lineages and functions, whereas γδ T cell metabolism is still poorly understood (70, 111). γδ T cells express mature γδTCR complex and undergo extensive proliferation comparable to αβ T cells (112–114). Unlike conventional T cells that exit the thymus as naïve T cells and further differentiate in peripheral organs upon activation, a large portion of γδ T cells commits to producing either IL-17 or IFN-γ during the development in the thymus, called the IL-17-producing γδ T cell (γδT17) and the IFN-γ-producing γδ T cell (γδT1), respectively (115–117). The two have intrinsically different metabolic requirements. Notably, the TCRγδ signaling appears to be more favorable for the metabolic transition of thymic γδ progenitors to γδT1 cells that are highly glycolytic. In contrast, γδT17 mainly engages OXPHOS (118). The metabolic dichotomy established in the thymus has a significant impact on the expansion and function of γδT1/17 cells, which could be used in tumor and autoimmune disease therapies (118–123). Yang et al. demonstrated that both γδT1 and γδT17 cells require mTORC1 for proliferation and survival, whereas mTORC2 is only essential for γδT17 cells. In Raptor KO mice, γδT17 differentiation was impaired due to suppressed glycolysis. In contrast, mTORC2 potentiated γδT17 induction by reducing mitochondrial ROS production (122). Moreover, nitric oxide synthase 2 (NOS2) deficient mice exhibited substantially reduced glycolysis and proliferation of γδ T cells (124). Glutaminase 1-mediated glutaminolysis was aberrantly activated and promoted γδT17 differentiation, thereby resulting in the development and immune imbalance of psoriasis (125). A recent study revealed a key role of c-Maf in regulating the function of γδT17 effectors through IDH2-mediated metabolic reprogramming (126). In conclusion, thymocyte development is orchestrated by these key metabolic regulators in the thymus.

## Thymocytes return to a resting state from the small DP stage to CD4 or CD8 SP stage

After massive proliferation, DP thymocytes return to a resting state and initiate rearrangement of the Tcra gene for positive and negative selection. When quiescence occurs along a continuum as thymocytes differentiate from DP cells into SP cells, glucose metabolism must drastically decrease and revert to mitochondrial oxidative metabolism for maximal ATP generation (10, 127). Key metabolic regulators, such as c-Myc and HIF1α, need to be downregulated to safeguard the transition from the DP blast to the resting small DP stage (77, 84, 128). Moreover, the rise in metabolic activity in SP cells after the DP late stage could be due to functional TCR signaling that promotes the restoration of sensitivity to cytokines that fine-tune cellular metabolism (129, 130).

Although apoptosis of those DP cells that fail to be selected is crucial for thymocyte maturation program, it is also essential for pre-selection thymocytes to maintain a relatively low metabolic state to survive long enough waiting to be tested for their responses to self-peptide/MHC. Retinoid-related orphan nuclear receptor γt (RORγt) belongs to the nuclear hormone receptor superfamily of transcription factors and serves as a signaling node to connect lipid metabolism, inflammation, and immune cell responses (131). It has been demonstrated that RORγt expression reduces the abundance of cytokine receptors of the common chain (γc) and suppresses cellular metabolism and mitochondrial biogenesis in preselection DP thymocytes. DP thymocytes lacking RORγt exhibit features identical to persistent T cell expansion (132). O-linked β-N-acetylglucosamine protein O-GlcNAcylation shows a concomitant dynamic regulation with consecutive phases of T cell development and is controlled by Notch, c-Myc, and the T cell antigen receptors (133). O-GlcNAc transferase (OGT) acts as a signaling hub to integrate thymocyte responses to developmental stimuli through modifying changes in glucose and glutamine supply (134). An interesting finding has been shown that Ogt^-/-^CD4^cre/+^ mice had normal numbers of DP thymocytes but failed to differentiate into mature CD4+ or CD8+ SP thymocytes (134, 135). Pyruvate dehydrogenase (PDH) is required for thymocytes to regulate metabolic processes such as the tricarboxylic acid (TCA) cycle, redox homeostasis, glutathione levels, and pyruvate accumulation (136–138). Thymocytes can oxidize more glucose in the TCA cycle through PDH, and loss of PDH decreases the number of DP cells (139). Heme (iron-protoporphyrin IX) is an essential cofactor and signaling molecule involved in a vast array of biological processes, including cellular metabolism (140). Philip et al. reported a surprising finding that feline leukemia virus subgroup C receptor (FLVCR), the major facilitator superfamily (MFS) metabolite transporter and the heme exporter, was required for thymocyte development beyond the DP stage by supporting heme metabolism (141, 142). With FLVCR deletion during the DN stage, mice had a complete block in αβ T cell development at the DN-DP transition, whereas loss of FLVCR at the DP stage affects peripheral T cell proliferation and apoptosis (143).

THEMIS, a T cell specific protein that is highly expressed in DP cells, directly regulates the catalytic activity of SHP-1 by promoting ROS-mediated oxidation of the SHP-1 active site cysteine to facilitate thymic positive selection (144). N-linked glycosylation (NLG) also has an important impact on thymocyte selection. On the one hand, NLG negatively regulates the activity of high-affinity TCR, allowing thymocytes with these receptors to survive during negative selection. On the other hand, NLG increases expression of the CD4/CD8 co-receptors, allowing thymocytes with low-affinity TCR to survive during positive selection (145, 146).

Thymocyte egress is a crucial determinant factor of T cell homeostasis and adaptive immunity (147). Recently, protein geranylgeranyltransferase type I catalytic β-subunit (Pggt1b) has been shown to be involved in thymocyte egress by maintaining mevalonate metabolism–fueled posttranslational modification. Du et al. demonstrated that the expression of Pggt1b was upregulated in SP cells in comparison with DP cells, and deletion of Pggt1b impaired thymocyte egress, resulting in severe peripheral T cell lymphopenia but the accumulation of mature SP thymocytes in the thymus (148). Ultimately, CD4+ or CD8+ single-positive cells exit the thymus and circulate to peripheral organs, such as the spleen and lymph node.

The development of invariant natural killer T (iNKT) cells is more sensitive to changes in mitochondrial electron transport chain function than conventional αβT cells (149). There have been several informative reviews recently, so we won’t go over those details here (150–152).

## Conclusion and perspective

**Figure 1:**
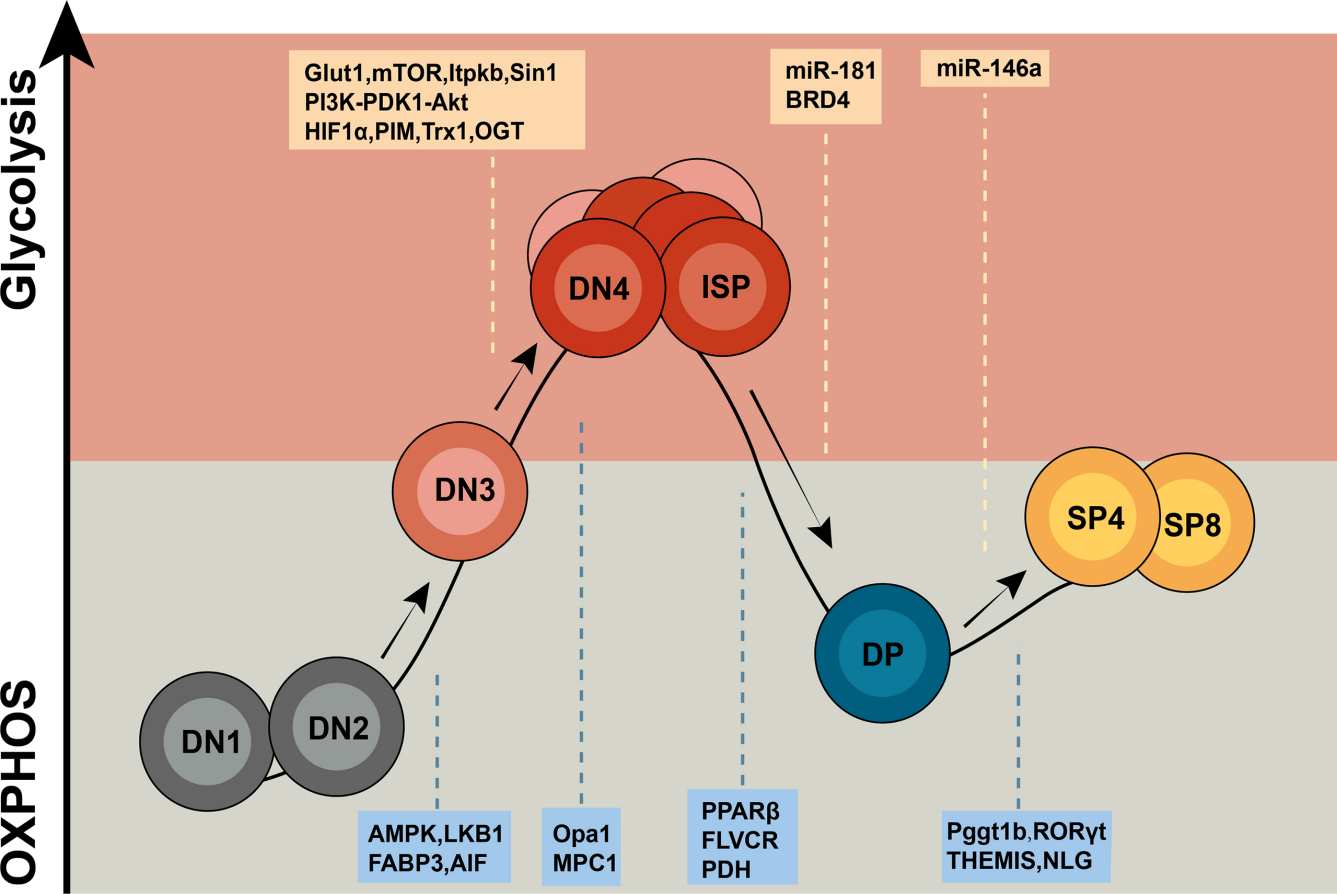
Overview of metabolic regulators in T cell development. Thymocytes display distinct metabolic profiles depending upon their states of development. DN1 and DN2 T cells are metabolically quiescent and adopt a basal level of nutritional intake, relying on OXPHOS as the primary approach of ATP production. Upon proliferation, T cells from the DN3b stage to early DP stage shift to a state of metabolic activation characterized by incremental nutrient uptake and elevated glycolysis. Then, T cells return to a resting state from the small DP stage to CD4+/CD8+ SP stage. The letters in the yellow box represent glycolysis regulators, and the letters in the blue box represent OXPHOS regulators.

**Figure 2:**
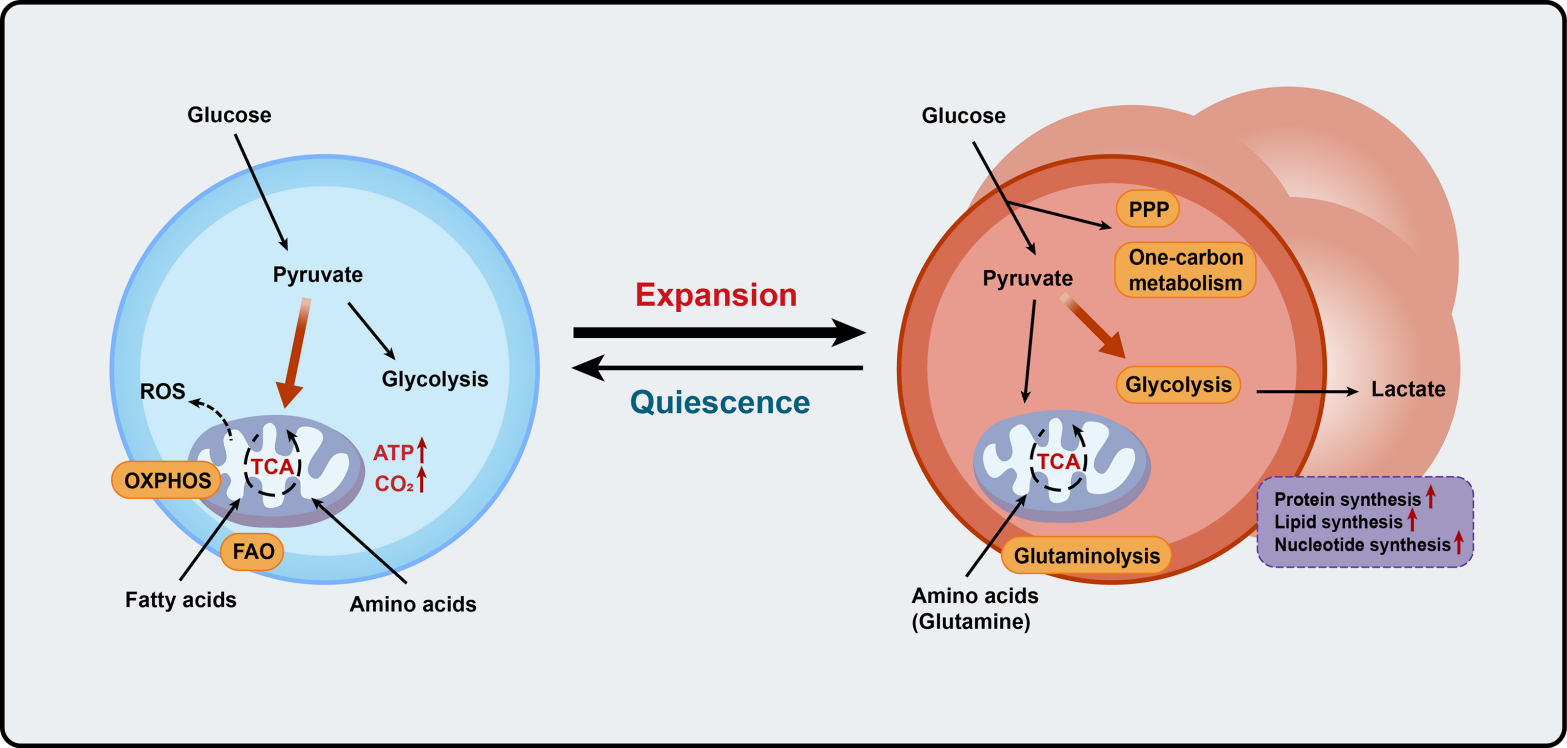
Metabolic programs match expansion demands of thymocytes. Blue cells on the left represent the quiescent thymocytes, and red cells on the right represent proliferative thymocytes. OXPHOS, oxidative phosphorylation; FAO, fatty acid oxidation; ROS, reactive oxygen species; ATP, adenosine triphosphate; PPP, pentose phosphate pathway; TCA, the tricarboxylic acid.

In the past few years, extensive studies have largely focused on the metabolic regulation of T cell differentiation and responses. However, our knowledge of how cellular metabolism modulates thymocyte maturation in response to developmental signaling pathways and microenvironmental cues remains limited. Emerging studies using gene knockout mouse models have demonstrated that key metabolic regulators and enzymes are involved in different stages of thymocyte maturation by modulating the metabolic pathways and signaling networks to match the specific functional requirements of the stage (**Figures 1** and **2**). Recent progress in single-cell metabolomics, CRISPR/Cas9, and spatially resolved metabolomics will continue to add valuable findings to this field (10, 153). Future work on the molecular mechanisms of cell context-dependent regulation of these metabolic processes will not only enhance our understanding of the interplay between cellular metabolism and T cell developmental programs but also provide potential novel therapeutic strategies to modulate immune responses.

## Author contributions

JH and BZ: conceptualization and guidance. MZ wrote and edited the manuscript. All authors contributed to the article and approved the submitted version.

## Funding

This study was supported by the National Natural Science Foundation of China (grant numbers 82170795 and 82100949), and the Outstanding Young Investigator of Hunan Province (2022JJ10094).

## Conflict of interest

The authors declare that the research was conducted in the absence of any commercial or financial relationships that could be construed as a potential conflict of interest.

## Publisher’s note

All claims expressed in this article are solely those of the authors and do not necessarily represent those of their affiliated organizations, or those of the publisher, the editors and the reviewers. Any product that may be evaluated in this article, or claim that may be made by its manufacturer, is not guaranteed or endorsed by the publisher.
